# AFM-based nanoindentation indicates an impaired cortical stiffness in the AAV-PCSK9^DY^ atherosclerosis mouse model

**DOI:** 10.1007/s00424-022-02710-x

**Published:** 2022-06-01

**Authors:** Leonie Achner, Tobias Klersy, Benedikt Fels, Tobias Reinberger, Cosima X. Schmidt, Natalie Groß, Susanne Hille, Oliver J. Müller, Zouhair Aherrahrou, Kristina Kusche-Vihrog, Walter Raasch

**Affiliations:** 1 Institute of Experimental and Clinical Pharmacology and Toxicology, University of Lübeck, Ratzeburger Allee 160, 23538 Lübeck, Germany; 2 Institute for Physiology, University Lübeck, Lübeck, Germany; 3 DZHK (German Centre for Cardiovascular Research), partner site Hamburg/Kiel/, Lübeck, Germany; 4 Institute for Cardiogenetics, University Lübeck, Lübeck, Germany; 5 Institute of Neurobiology, University of Lübeck, Lübeck, Germany; 6 Institute for Experimental Dermatology (LIED), University of Lübeck, Lübeck, Germany; 7 Department of Internal Medicine III, University Hospital Schleswig-Holstein, Campus Kiel, Kiel, Germany; 8 CBBM (Centre for Brain, Behavior and Metabolism), University of Lübeck, Lübeck, Germany

**Keywords:** Atherosclerosis, AAV-PCSK9^DY^ mouse model, Atomic force microscopy, Cortical stiffness, Endothelial dysfunction

## Abstract

Investigating atherosclerosis and endothelial dysfunction has mainly become established in genetically modified ApoE^−/−^ or LDL-R^−/−^ mice transgenic models. A new AAV-PCSK9DY^DY^ mouse model with no genetic modification has now been reported as an alternative atherosclerosis model. Here, we aimed to employ this AAV-PCSK9^DY^ mouse model to quantify the mechanical stiffness of the endothelial surface, an accepted hallmark for endothelial dysfunction and forerunner for atherosclerosis. Ten-week-old male C57BL/6 N mice were injected with AAV-PCSK9^DY^ (0.5, 1 or 5 × 10^11^ VG) or saline as controls and fed with Western diet (1.25% cholesterol) for 3 months. Total cholesterol (TC) and triglycerides (TG) were measured after 6 and 12 weeks. Aortic sections were used for atomic force microscopy (AFM) measurements or histological analysis using Oil-Red-O staining. Mechanical properties of in situ endothelial cells derived from ex vivo aorta preparations were quantified using AFM-based nanoindentation. Compared to controls, an increase in plasma TC and TG and extent of atherosclerosis was demonstrated in all groups of mice in a viral load-dependent manner. Cortical stiffness of controls was 1.305 pN/nm and increased (10%) in response to viral load (≥ 0.5 × 10^11^ VG) and positively correlated with the aortic plaque content and plasma TC and TG. For the first time, we show changes in the mechanical properties of the endothelial surface and thus the development of endothelial dysfunction in the AAV-PCSK9^DY^ mouse model. Our results demonstrate that this model is highly suitable and represents a good alternative to the commonly used transgenic mouse models for studying atherosclerosis and other vascular pathologies.

**Supplementary information:**

The online version contains supplementary material available at 10.1007/s00424-022-02710-x.

To induce atherosclerosis in mice, atherogenic genetically modified transgenic ApoE^−/−^, LDL-R^−/−^, or ApoE/LDL-R^−/−^mice are needed. Very recently, a PCSK9-AAV [pro-protein convertase subtilisin/kexintype 9 (PCSK9) adeno-associated virus (AAV)] was reported as a model for atherosclerosis that does not require genetic modification [3]. A single injection of AAV-PCSK9^DY^ doubled the serum cholesterol concentration after just 30 days and it remained steadily increased over a period of 1 year [3, 32]. This hyperlipidemia was much more pronounced when AAV-PCSK9^DY^ mice were additionally fed a high-fat diet, as the plasma cholesterol concentration then increased to approx. 1200 mg/dl (compared to 320 mg/dl with the Chow diet) [32]. It might also be possible to accelerate atherosclerosis development in mice by overexpressing AAV-mediated PCSK9^DY^ and performing partial carotid ligation [23]. As a result of the hyperlipidemia after injecting AVV-PCSK9^DY^, the mice developed the full picture of atherosclerosis (development of plaques, macrophage infiltration, formation of foam cells, fibrosis, and formation of lesions) in a dose-dependent manner [3, 18, 30, 32, 39]. A single AVV-PCSK9^DY^ injection was also demonstrated to induce vascular calcification in wild-type C57BL/6 J mice [16]. Despite AVV-PCSK9^DY^ injection, however, this method does not cause any additional side effects (e.g., liver toxicity or inflammation) or pose an increased biological safety risk [13, 32]. Indeed, this method is fast, cost-effective, and versatile and therefore constitutes an important tool in atherosclerosis research [13].

To the best of our knowledge, no papers have been published yet demonstrating endothelial functional impairments in the AAV-PCSK9^DY^ model in contrast to genetically modified models of atherosclerosis. Arterial properties are usually measured either on the macroscale by determining the elastic modulus of an artery, which is based on the quotient of stress and strain derived from tensile testing, or on the microscale to determine layer-specific mechanics at the cellular level [22]. Thus, in arteries from both ApoE^−/−^ and ApoE/LDL-R^−/−^ mice on a high-cholesterol diet, dilation to ACh was decreased [25]. Moreover, stiffness of aortic arch and carotid arteries increased in ApoE^−/−^ mice being fed a high-fat diet, which was determined by either echocardiography or an inflated in situ system [6, 37]. While significant emphasis has been placed on macroscale measurements of vessel mechanics, less is known about changes in the mechanical properties of the individual arterial layers or the extracellular matrix within these layers [22]. New techniques that ensure such approaches include micropipette aspiration and the atomic force microscopy (AFM)-based nanoindentation [22]. By applying Raman- and AFM-based detection methods, an increased intracellular lipid content and elevated cortical stiffness/elasticity were shown in ApoE/LDL-R^−/−^ aortas, demonstrating a direct link between endothelial dysfunction, the biochemical composition, and the nanomechanical properties of endothelial cells (ECs) [27].

Hence, we aimed here to investigate endothelial stiffness in AAV-mediated mice overexpressing PCSK9^DY^ by employing the AFM technique, which is the hallmark method for investigating endothelial dysfunction. According to AFM-based nanoindentation measurements, layer-specific mechanical properties can be determined [28] and can be performed ex vivo, as described elsewhere [10, 19, 20, 38]. The mechanical stiffness of the ex vivo ECs provides information about structural and functional differences between the aforementioned groups. It can be seen as the hallmark for proper endothelial (dys)function. Therefore, we performed AFM-based nanoindentation measurements in in situ ECs derived from aortic segments of AAV-PCSK9^DY^-treated mice (0 × , 0.5 × , 1 × , and 5 × 10^11^ VG) to induce atherosclerostic lesions in male C57BL/6 N mice. Atherosclerosis development was confirmed by measuring plasma levels of cholesterol and lipids as well as by histologically evaluating plaque content.

## Methods

### AAV vector production and purification

AAV serotype 8 vectors for expression of the murine D377Y-PCSK9 cDNA (AAV-PCSK9^DY^) were produced as previously described [5] by using the two-plasmid method and cotransfecting AAV/D377Y-mPCSK9 (gift from Jacob Bentzon; Addgene plasmid # 58,376) [3] together with the helper plasmid pDP8 [35] in HEK293T cells using polyethylenimine (Sigma-Aldrich). AAV vectors were purified using iodixanol step gradients and titrated as previously described [21]. In the following, we denote this vector as AAV-PCSK9^DY^.

### Animals

All animal care and experimental procedures were conducted in accordance with the NIH guidelines for care and use of laboratory animals and were approved by the local animal ethics committee (Ministerium für Energiewende, Landwirtschaft, Umwelt, Natur und Digitalisierung des Landes Schleswig–Holstein, Germany) under the application number V241-60,532/2017(75–7/19, 85–9/18). The results of all studies involving animals are reported in accordance with the ARRIVE guidelines [31]. The group sizes of *n* = 6 in each group were assessed by a power analysis (corrected *α* = 0.008, power 80%) by considering changes in total cholesterol plasma concentration [32]. In total, 24 mice were used for this study. Eight-week-old male C57BL/6 N mice (Charles River) were kept in standard housing in groups of four individuals randomized by weight. Mice had ad libitum access to standard chow diet for a 2-week habituation period before starting the study and receiving the AAV-mPCSK9^DY^ injections. One mouse in the 1 × 10^11^ VG group died for unknown reasons shortly before the study ended and one mouse from group 3 (5 × 10^11^ VG) was consistently excluded from the evaluations because it became ill.

### AAV-PCSK9^DY^ administration as a spontaneous atherosclerosis model

Mice were injected with AAV-PCSK9^DY^ (0.5 or 1 or 5 × 10^11^ VG in 100 µl) or with 100 µl saline via the tail vein at an age of 10 weeks. With regard to the virus load, we were guided by data from previous studies [3, 5]. Afterwards, mice were fed a Western diet (WD, EF TD88137 mod. + 1.25% cholesterol, ssniff Spezialdiäten GmbH) for 3 months. After 6 and 12 weeks, approx. 100 µl EDTA-blood was withdrawn retro-orbitally. After 12 weeks on the WD, mice were given an overdose of isoflurane by inhalation and sacrificed by cervical dislocation. Aortas (from the aortic root to the iliac bifurcation) were removed, after which fat and connective tissue were cleaned and then immediately used for AFM measurements or fixed in 4% paraformaldehyde solution for histological analysis (plaque lesion analysis) or were snap frozen and stored at − 80 °C (molecular biological studies).

### Body weight and body composition

Body weight was monitored 2–3 times per week. Body composition was measured at week 11 with the Minispec BCA analyzer (LF-110, Bruker) 2 h after the mice were transferred to the room to acclimatize to the environment. For the measurements, mice were put into the restrainer, which was then placed in the analyzer [17].

### Stiffness measurements by AFM

The mechanical properties, i.e., function of vascular ECs, were determined in the aortas of the treated mice 24 h and 48 h after aortas were prepared by using the AFM-based nanoindentation measurements as described elsewhere [10, 19, 20, 38]. In brief, a spherical AFM tip, mounted on the end of a highly flexible cantilever, is used as a mechanical sensor as it is gradually lowered onto the cell and indents the cell membrane upon contacting the upper surface. The resulting deflection of the cantilever, which serves as a soft spring, is measured via reflection of a laser beam from the gold-coated back side of the cantilever and can be directly related to the stiffness of the cell. Cantilever deflection can thus be plotted as a function of tip position along the *z*-axis. In order to quantify cell stiffness, these data are transformed into a force-versus-deformation curve, using the cantilever’s spring constant and the optical cantilever sensitivity. The slope of this force-distance curve then directly reflects the force (in nano Newton), here defined as stiffness, that must be exerted to indent the cell for a certain distance.

Mouse aortas were isolated and freed from surrounding tissue. A small patch (≈1 mm^2^) of the whole aorta was removed and attached on Cell-Tak (BD Biosciences, Bedford, MA, USA) coated slide, with the endothelial surface facing upward and cultured at 37 °C and 5% CO2 in minimal essential medium (MEM; Invitrogen, Life Technologies,Carlsbad, CA, USA), containing 10% fetal calf serum (FCS; PAA Laboratories, Pasching, Austria, 1% MEM vitamins (Biochrom, Ltd., Cambridge, UK), penicillin G (10,000 U/ml), streptomycin (10,000 mg/ml), and 1% MEM nonessential amino acids (MEM NEAA; Gibco, Life Technologies) [24].

Stiffness measurements of living aortic ECs in situ were conducted with an atomic force microscope (Nanoscope Multimode8 AFM, Bruker). All experiments were performed in HEPES-buffered solution [standard composition in mM: 140 NaCl, 5 KCl, 1 MgCl2, 1 CaCl2, 5 glucose, 10 HEPES (N-2-hydroxyethylpiperazine-N′-2-ethanesulfonic acid), pH 7.4, 37 °C]. For AFM measurements, a cantilever (Novascan Technologies, Boone, NC, United States) with a mounted spherical tip (diameter 10 μm) and a nominal spring constant of 30 pN/nm was used. A maximal loading force of 4.5 nN was applied and force-distance curves were created in eightfold replicates on 44–62 cells in each mouse. These AFM data were collected with Research NanoScope Software (version 9.20 (Bruker 2016); 2) and stiffness values were calculated from force-distance curves using the Protein Unfolding and Nano-Indentation Analysis Software Punias 3D (Version 1.0; Release 2.3; Copyright 2009).

### Histological analysis

Atherosclerotic lesions were quantified in the aortic root of the mice as described previously [1, 33]. For all atherosclerotic plaque analyses, investigators were blinded to the viral load. In brief, serial cross-Sects. (8- to 10-μm thick) were obtained, starting below the aortic root to the proximal aorta, below the aortic arch. The sections were stained using Oil Red-O (ORO) and the mean atherosclerotic lesion area was calculated from 8 to 10 sections at 40-μm intervals, starting at the appearance of at least two aortic valves until the aortic valves disappeared. Images were acquired using a Keyence microscope (BZ-X800). Sections were manually cropped using GIMP software, version 2.6 (The GIMP Development Team) to yield aortic root areas (see Supplementary Fig. S1). Areas of lesions and ORO-positive regions were determined using an in-house Python script (available on request). In brief, the Python package OpenCV (https://pypi.org/project/opencv-python/) was utilized to process images and to determine lesions based on color thresholds (e.g., reddish pixel for ORO). The ratio of ORO-positive lesions in each animal was determined as the percentage lesion area, normalized to the total area of the aorta.

### Cholesterol (TC) and triglyceride (TG) analyses

Plasma was prepared by centrifugation. Plasma concentrations of TC and TG were analyzed in the mice as described previously [1] to study whether an increasing viral load affects lipid profiles.

### Blood cytokine analysis

The LEGENDplex™ Mouse Inflammation Panel (BioLegend, #740,446) is a multiplex assay and was used to quantify 13 mouse cytokines in 10 µl serum, including IL-1α, IL-1β, IL-6, IL-10, IL-12p70, IL-17A, IL-23, IL-27, MCP-1, IFN-β, IFN-γ, TNF-α, and GM-CSF. The assay was performed according to the manufacturer’s instructions.

### Statistical analysis

GraphPad Prism 8.0 (La Jolla, USA) was used for statistical analysis. All data were checked for outliers and were tested for Gaussian distribution and variance homogeneity. One-way ANOVA followed by Tukey’s multiple comparisons test was used for comparing the four different groups, assuming a Gaussian distribution and variance homogeneity. Alternatively, if the Gaussian distribution was not given, we used Kruskal Wallis Test followed by Dunn’s multiple comparisons test. When comparing data including more than one variable, we used 2-way ANOVA followed by Tukey’s or Sidak’s multiple comparisons test. Correlation analyses were performed by applying the 2-tailed Pearson test. A *P*-value < 0.05 was considered statistically significant. In box graphs (representing the 25th to 75th percentiles) with whiskers (representing the maximum and minimum values), both the individual data and the medians are presented. In the line graphs, means ± SDs are depicted.

## Results

### Influence of AAV-PCSK9^DY^ plus Western diet on growth of mice

**Fig. 1:**
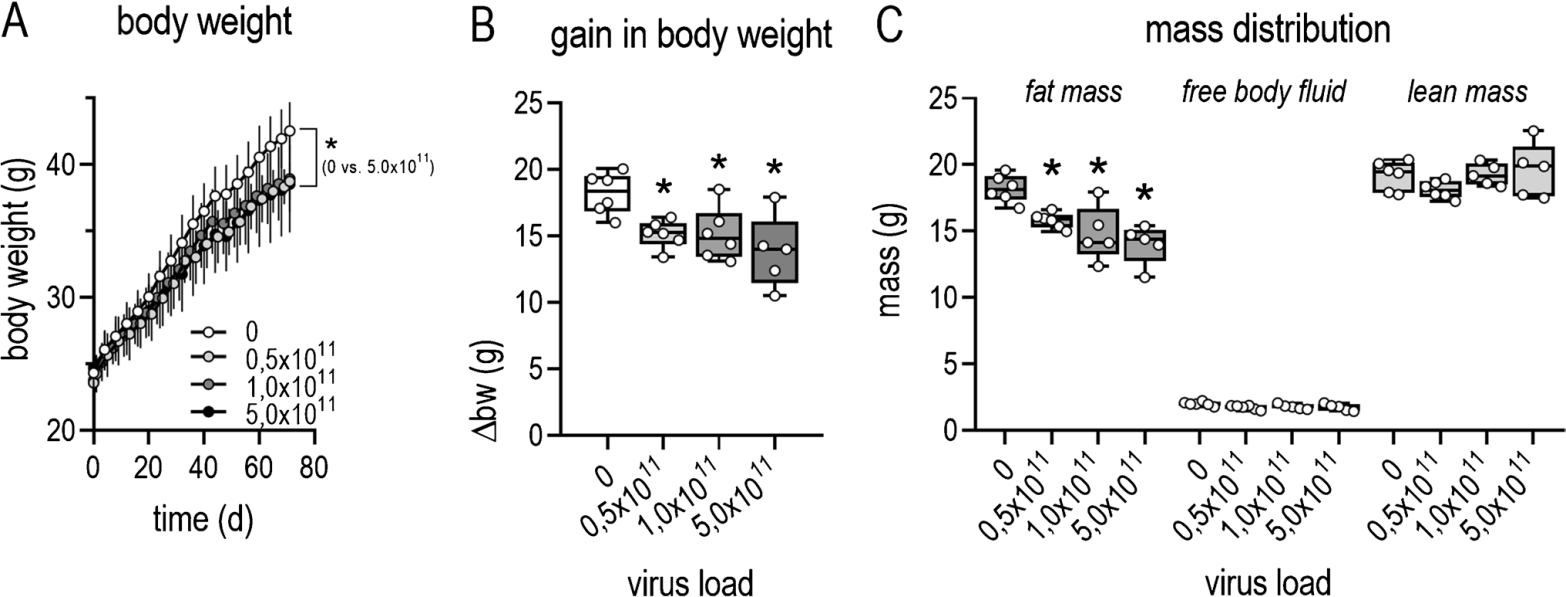
Growth of WD-fed mice in dependency of the PCSK9 viral load. **A** development of body weight within the 12-week treatment period; means ± SDs (*n* = 5–6), 2-way ANOVA followed by Tukey’s multiple comparisons test [time: *F* = 277, *P* < 0.0001, treatment: *F* = 2.9, *P* = 0.068, interaction (time × treatment): *F* = 216, *P* < 0.0001]; **B** gain in body weight (1-way ANOVA [*F* = 5.568, *P* = 0.0065) followed by Tukey’s multiple comparisons test); **C** mass distribution after 12 weeks (Kruskal Wallis Test (fat mass *P* = 0.0028; free body fluid *P* = 0.0906; lean mass *P* = 0.359) followed by Dunn’s multiple comparisons test was calculated as Gaussian distribution of the values was not given; the median is depicted in box blots; the box extends from the 25th to 75th percentiles and the whiskers go down to the smallest value and up to the largest; *n* = 5–6; *P < 0.05 vs 0

Body weight of the mice did not differ before injecting the virus. In response to virus injection plus the 12-week WD-feeding regimen, the body weight was almost doubled. This impression is confirmed by 2-way ANOVA testing, as the factor time became highly significant. Moreover, 2-way ANOVA indicated a statistical significance in relation to the interaction time × treatment. This was specified in the post-analysis (Tukey’s multiple comparisons test) and became apparent by the significant difference between 0 and 5 × 10^11^ vg (Fig. 1A). To confirm the weight regulatory effect of viral load, we additionally evaluated the weight gain over time, which is defined as the difference between final and initial weight. Thus, the gain in body weight was 35% lower in the group treated with 5 × 10^11^ VG than in controls (Fig. 1B). Accordingly, mass distribution was diminished by increasing viral loads, where, in particular, the lean mass remained stable while the fat mass was decreased in the 1 × and 5 × 10^11^ VG groups (Fig. 1C).

### Influence of AAV-PCSK9^DY^ plus Western diet on atherosclerotic lesions

**Fig. 2:**
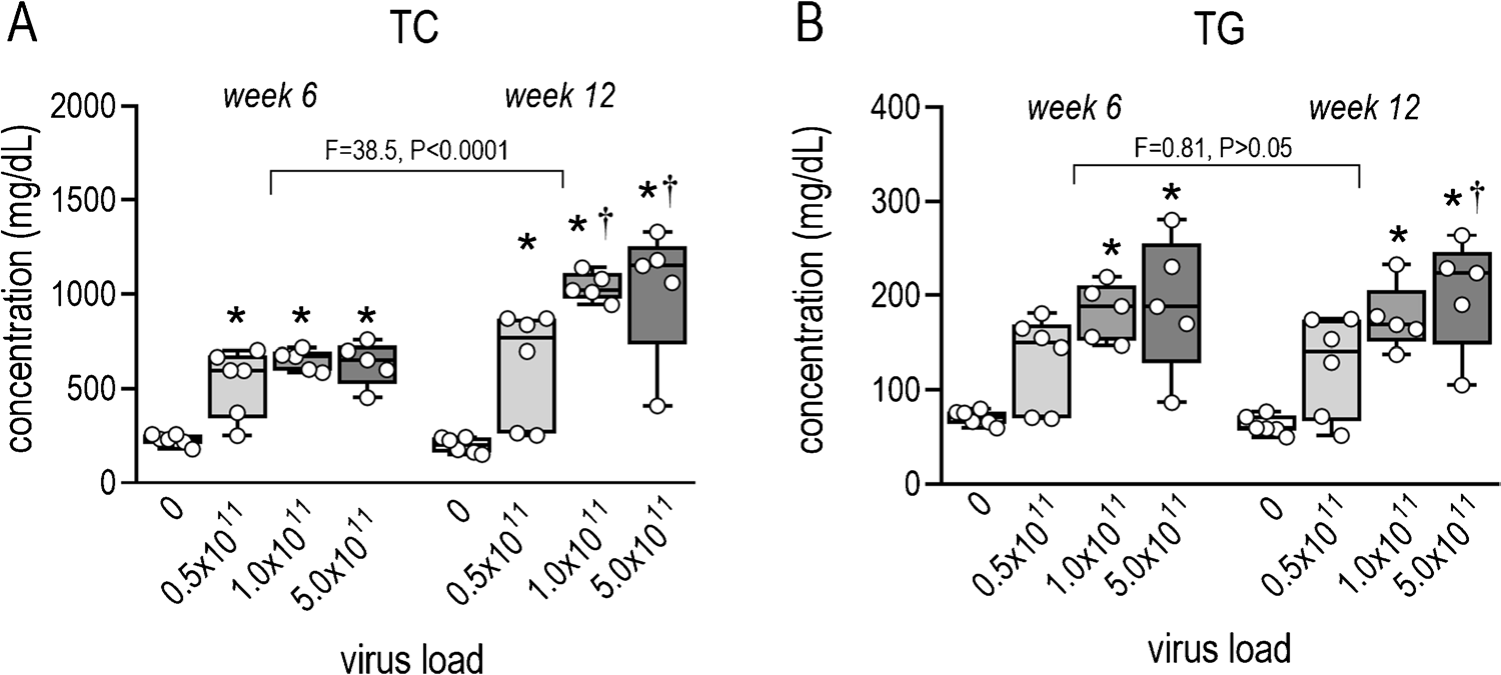
Plasma concentrations of cholesterol (**A**) and triglycerides (**B**) after 6 and 12 weeks in WD-fed mice in dependency of the PCSK9^DY^ viral load. Plasma cholesterol and triglycerides were calculated using by using 2-way ANOVA considering the factors time and viral load (cholesterol *F* = 17.75, *P* < 0.0001; TG *F* = 11.36, *P* = 0.0002) followed by Sidak’s multiple comparisons test. *n* = 5–6; the median is depicted in box blots; the box extends from the 25th to 75th percentiles and the whiskers go down to the smallest value and up to the largest. *P < 0.05 vs 0; †*P* = 0.05 vs. 0.5 × 10^11^

**Fig. 3:**
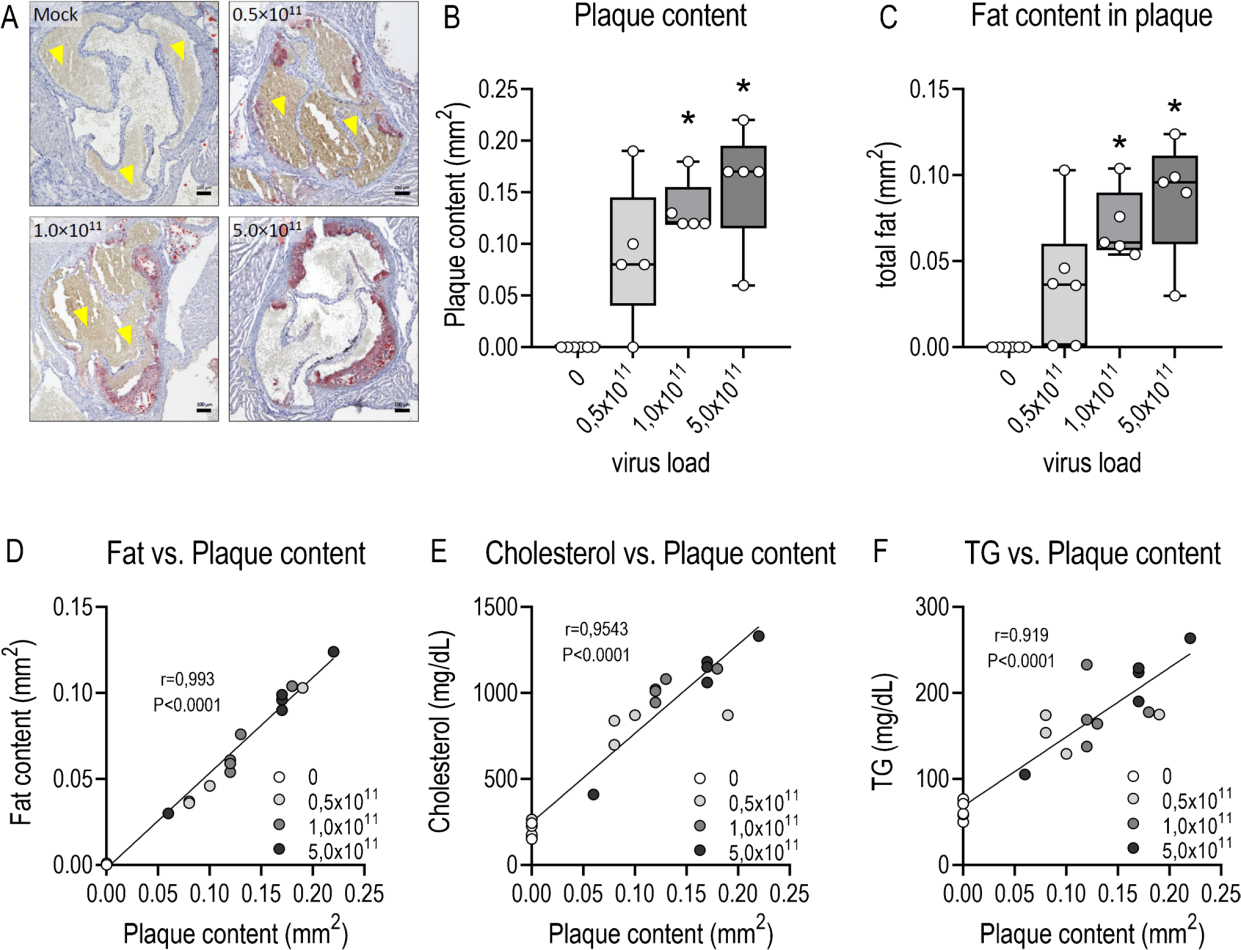
Histologic evaluation of aortic root of WD-fed mice in dependency of the PCSK9.^DY^ viral load. **A** depicts exemplary aortic segments of each treatment group upon Oil Red O staining (the brownish colorations marked with an arrow are blood residues in the samples and are evaluated as artifacts; scale bar: 100 µm); **B** quantitative evaluation of plaque content; **C** quantitative evaluation of fat content. A Kruskal Wallis test (plaque content *P* = 0.0044; fat content *P* = 0.0017) followed by Dunn’s multiple comparisons test was calculated; correlation analyses were performed by 2-tailed Pearson test; the median is depicted in box blots; the box extends from the 25th to 75th percentiles and the whiskers go down to the smallest value and up to the largest

Plasma cholesterol levels in control mice were similar at week 6 and 12. Cholesterol had more than doubled in PCSK9^DY^-treated mice after 6 weeks but had increased even more after 12 weeks (*F* = 38.5, *P* < 0.0001)), namely, by a factor > 3 in mice treated with 0.5 × 10^11^ VG and in fact by a factor of 5 in mice receiving 1 × or 5 × 10^11^ VG (Fig. 2A). No difference in TC level was observed between 1 × or 5 × 10^11^ VG. TGs similarly increased in response to PCSK9^DY^ although values did not rise more than threefold over controls after 12 weeks. In contrast to the cholesterol, plasma triglyceride levels did not differ between week 6 and 12 (Fig. 2B). Most cytokine levels were below the detection limit of the test used. Only in the case of IL1α, IL-6, and INF-γ could values sufficiently above the detection limit be obtained that could then be evaluated. However, the variability was too high and, at the same time, the number of measured values was too low, so that, contrary to our expectation, no differences in plasma levels could be detected as a function of viral load (Fig. S2A, S2B, S2C). ORO staining from aortic rings indicated atherosclerotic lesions in all groups of mice receiving PCSK9^DY^ (Fig. 3A). Compared to controls, plaques and fat content clearly increased, particularly by using the higher doses of 1 × or 5 × 10^11^ VG (Fig. 3B and C). Thus, plaque content perfectly correlated with fat content and almost perfectly with TC and TG levels (Fig. 3D, E and F).

### Influence of AAV-PCSK9^DY^ plus Western diet on endothelial/cortical stiffness

**Fig. 4:**
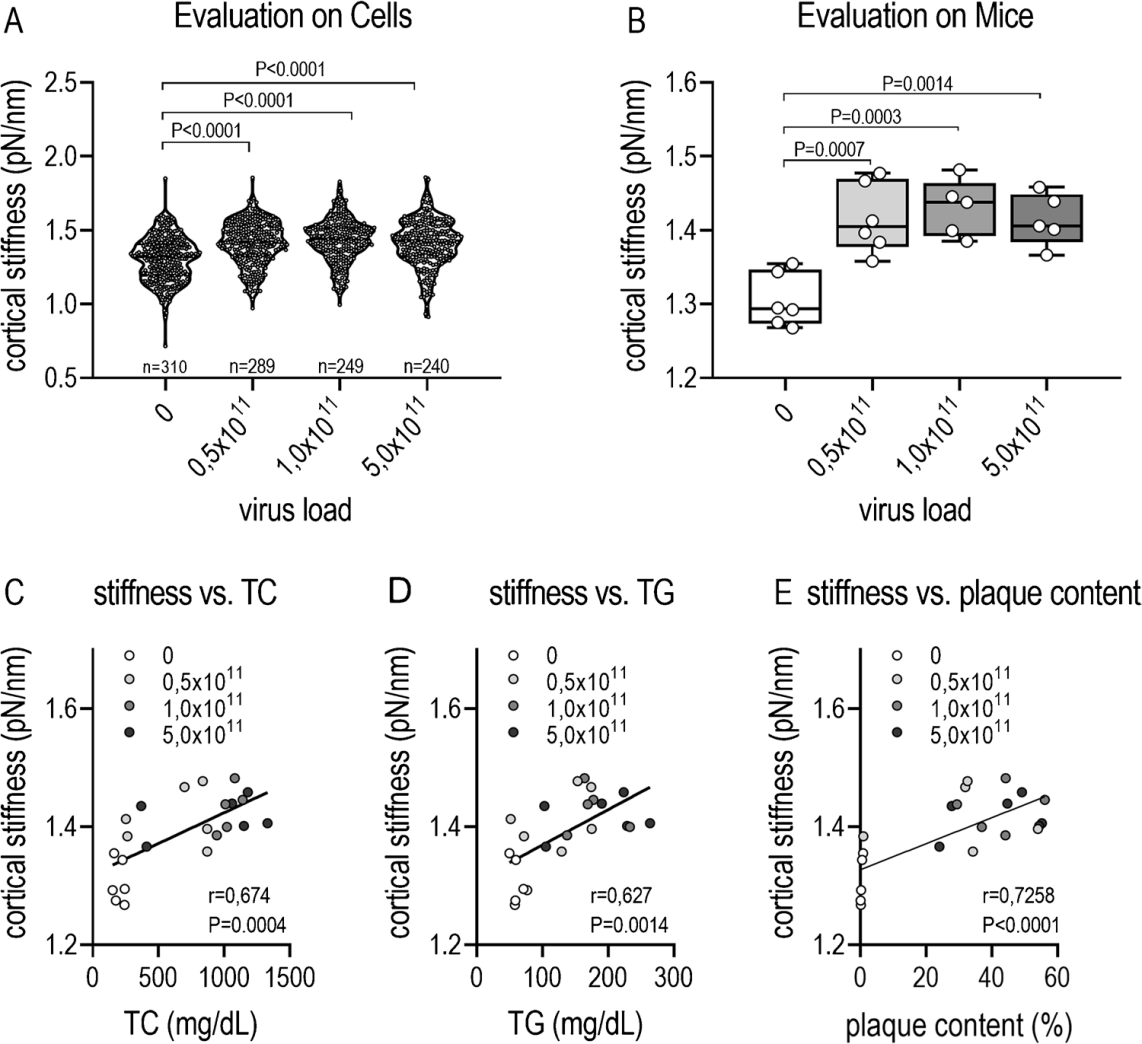
Cortical stiffness in WD-fed C57BL/6 N mice which were treated with AAV-PCSK9 (0, 0.5, 1 or, 5 × 10.^11^ VG). **A** Stiffness values were calculated in 44–62 cells of each mouse from force-distance curves in a blinded manner by using the PUNIAS 3D version 1.0 release 1.8 (http://punias.voila.net/); to detect statistical differences between groups, a Kruskal Wallis test (*P* < 0.0001) was calculated followed by Dunn’s multiple comparisons test; **B** evaluation considering aortic stiffness in individual mice; statistical differences between groups were detected by 1-way ANOVA testing (*F* = 12.24, *P* = 0.0001) followed by Tukey’s multiple comparisons test; values are depicted as box plots showing 25th and 75th percentile (box) and maximum/minimum values (whiskers). The horizontal black line depicts the median in each group; **C**–**E** Correlation analysis between cortical stiffness and cholesterol, triglycerides, or plaque content. Correlations between 2 factors were calculated by Pearson

As a functional parameter, the cortical stiffness of ECs was determined in a total of 240–310 ECs per group (approx. 50 ECs in one aorta of each mouse). Considering the statistical analyses of individual ECs, the cortical stiffness highly significantly increased depending on AVV-PCSK9^DY^ treatment without showing any genetic dose effect (Fig. 4A; controls 1.306 ± 0.149 pN/nm; 0.5 × 10^11^ VG 1.414 ± 0.145 pN/nm, 1 × 10^11^ VG 1.430 ± 0.143 pN/nm, 5 × 10^11^ VG 1.413 ± 0.157 pN/nm). Even when statistical analysis was based on individual mice of each group, the increase in stiffness upon PCSK9^DY^ treatment remained significant by also detecting no differences as a function of viral load (Fig. 4B; controls 1.305 ± 0.036 pN/nm; 0.5 × 10^11^ VG 1.416 ± 0.047 pN/nm, 1 × 10^11^ VG 1.430 ± 0.038 pN/nm, 5 × 10^11^ VG 1.414 ± 0.036 pN/nm). In accordance with histological examinations, cortical stiffness positively correlated with plasma levels of cholesterol and TGs (Fig. 4C and D). Moreover, we found a positive correlation between cortical stiffness and the aortic plaque content (Fig. 4E).

## Discussion

There is broad consensus that atherosclerosis results in vasomotor dysfunction, at least in part. This has mostly been demonstrated in different vessel segments from ApoE^−/−^, LDL receptor^−/−^, or apoE/LDL R^−/−^ mice fed with a high-fat diet, thus featuring hypercholesterolemia. Based on these genetic models, endothelium-dependent and -independent vascular functions were generally assessed with diverse macroscale mechanical tests (e.g., tensile testing) by measuring the vasoactive responses in arteries after administering norepinephrine, sodium nitroprusside, NO, serotonin, PGI2 and EDHF, or acetylcholine (ACh) [2, 4, 8, 9, 11, 12, 25, 40]. Such tensile testing suggested that stiffening may precede significant disease progression. Arteries are composed of intima, lamina, media, and adventitia, each with a distinct composition and function, thus raising the question as to which of these concentric layers is involved. However, when evaluating tensile tests in response to diverse vasoconstrictors or dilatators, arterial mechanics have been studied more widely on a macroscale, where properties of the media and adventitia dominate. Interestingly, it was shown that ECs are mechanosensitive to matrix stiffness and that increased intimal stiffness promotes endothelial dysfunction. Thus, it was concluded that the mechanical property of the intima is a key regulator for the progression of cardiovascular diseases. In this regard, AFM was found to be a valuable technique for measuring mechanical stiffness of the ECs to increase understanding of the functional role of the endothelium in vascular dysfunction in atherosclerosis. Considering the force curves generated by the force mode AFM operation, mechanical properties of EC can be measured using this microscale technique (reviewed in [22]. Using the AFM method, endothelial dysfunction was also demonstrated in ApoE/LDL-R^−/−^ mice, as stiffness increased in ECs from aortas of this transgenic model featuring atherosclerosis, thereby indicating a direct link between endothelial dysfunction, the biochemical composition, and the nanomechanical properties of ECs [27].

We pursued two main objectives in our study. First, we used an alternative to the experimental atherosclerosis models (see above) usually employed, namely, the AAV-PCSK9^DY^ model. Here, we clearly demonstrated that a single injection of AAV-PCSK9^DY^ markedly increased plasma levels of cholesterol and triglycerides (over the 12 weeks of experimentation) and induces atherosclerotic plaques. All atherosclerosis-related parameters correlated perfectly with each other, clearly confirming development of atherosclerosis. Thus, we were able to confirm previous results from others also showing hypercholesterolemia, hyperlipidemia, and development of atherosclerotic lesions in response to AAV-PCSK9^DY^ and high-fat/high-cholesterol feeding [3, 5, 32]. A viral load of 1.0 × 10^11^ VG is assessed to be high enough to induce endothelial dysfunction and atherosclerotic lesions as the higher VG dose of 5.0 × 10^11^ did not further enhance cortical stiffness, plaque content, or plasma cholesterol and triglyceride levels.

Secondly, we did not characterize vascular dysfunction via vasorelaxation in response to Ach, for example, as is usually done, but we instead characterized vascular dysfunction by using the AFM technique, which addresses endothelial stiffness as a hallmark for both proper endothelial function and dysfunction. In this regard, stiffness of aortic smooth muscle cells was reported to be higher in spontaneously hypertensive rats than in Wistar-Kyoto normotensive controls by using the AFM technique [34]. In contrast to Sehgal et al. [34], we did not use primary VSMCs following isolation protocols for AFM measurements in our study, but rather living in situ ECs derived from aorta preparations, in contrast to previously published protocols [20], which mimics the in situ situation much better since the ECs may have been altered by cultivation. Thus, the AFM was found to be an experimental technique that not only can assess individual cells using high-resolution microscopy under physiological conditions but also can quantitatively determine the mechanical properties at the single-cell level [36]. Considering our aim to investigate whether endothelial cell mechanics are affected in AAV-mediated mice overexpressing PCSK9^DY^ by using the AFM technique, we have clearly demonstrated here that the mechanical stiffness of the endothelial cortex is indeed enhanced. Our conclusion that this vascular dysfunction is due to atherosclerotic lesions can be inferred from the good correlations between the endothelial stiffness and the atherosclerotic plaque load or the plasma cholesterol levels. In confirmation of our observations, the AFM technique has also been used in previous studies in atherosclerotic models except for the AAV-PCSK9^DY^ model, showing that (1) short-term plasma dyslipidemia is sufficient to induce significant *endothelial* stiffening of intact aortas and strongly exacerbate endothelial stiffening in the aortic arch in animals on a low-fat diet [26]; (2) in atherosclerotic plaques and fibrous caps, the lipid-rich areas dropped in comparison to the stiffer, surrounding area of the plaque in brachiocephalic arteries of ApoE/LDL-R^−/−^ mice [28]; and (3) that cortical stiffness/elasticity was elevated in ApoE/LDL-R^−/−^ aortas, thus confirming a direct link between endothelial dysfunction, the biochemical composition, and the nanomechanical properties of ECs [27].

Mechanical stiffening of the endothelial cortex is multifactorial. Among others, it depends on the insertion and/or activity of specific membrane proteins, e.g., ENaC (epithelial Na channel), ion composition of the extracellular fluid, and the polymerization state of the cortical actin [14, 15, 29, 38]. However, the change between a soft and stiff endothelial surface is physiologically important as it is a reaction, adaptation, and response to different conditions, making the endothelial surface an important regulatory hub for overall vascular function. Taking all this into account, it is obvious that the flexibility of the endothelial cell mechanics is limited. As demonstrated, e.g., ion channels, the cell can reach a “saturated” condition. This means that it cannot be stiffer (or softer) to fulfill its function. Stiffening above a (cell specific) threshold would end in complete dysfunction or even cell death. This is what we observed in the present study: AAV-mediated overexpression of PCSK9^DY^ with the lowest viral dose (0.5 × 10^11^ VG) leads to a stiff cortex which could not be further augmented with higher viral doses, indicated the max level of stiffness in the system. In other words, we hypothesize that already with the lowest viral dose, all mechanisms leading to an increase in mechanical stiffening are initiated (membrane insertion of proteins, expression of linker proteins, polymerization of actin). In the past, we have made similar observations, e.g., the membrane insertion of endothelial ENaC due to non-laminar shear stress compared to laminar shear stress [7]. In a follow-up study, exactly these processes are studied at the moment in vitro and ex vivo.

Also in the past, we have observed slight differences in the cortical stiffness of ex vivo endothelial cells, which might be caused by undefined variations in mouse strains and lab conditions (chow, stress factors, handling). This is not the case for endothelial cells in vitro, e.g., HUVEC or EAhy.926, where the absolute values are highly stable and comparable between different experimental series. We found in this study values of 1.306 ± 0.149 pN/nm in C57/BL/6 N mice and of 1.430 ± 0.143 pN/nm (+ 10% vs. C57/BL/6 J), while Maase et al. [27] detected in C57/BL/6 J 1.19 ± 0.034 pN/nm and in ApoE/LDL-R^−/−^: 1.38 ± 0.028 pN/nm (+ 15% vs. C57/BL/6 J) and Jeggle et al. [19] in C57/Bl6 1.7 ± 0.08 pN/nm. By comparing the absolute values between the AAV-system with ApoE/LDL-R^−/−^ mice, it seems that the latter is more effective in terms of cortical stiffening. However, this could also be due to many other conditions. However, we are convinced that the difference between control and treated group is way more important than the absolute values. Based on these facts, we prefer to compare the amplitude of the variation (in %), instead of the absolute values.

In conclusion, the AVV-PCSK9^DY^-based method is an ideal experimental atherosclerosis model for inducing atherosclerotic lesions in transgenic mice. This approach avoids the need for costly breeding of double transgenic animals to combine the desired transgene with an ApoE^−/−^ or ApoE/LDL-R^−/−^ background. Although we identified a viral load of 1 × 10^11^ VG to be high enough to induce endothelial dysfunction and atherosclerotic lesions, next, we will perform studies by using 2 × 10^11^ VG for safety reasons due to manufacturing-related batch fluctuations in virus production. The AFM is an elegant technique for functionally confirming the development of atherosclerosis in addition to the histological findings and plasma analyses of cholesterol and lipids.

## Supplementary information

Below is the link to the electronic supplementary material.

Supplementary file1 (PDF 784 KB)

## Data Availability

The data that support the findings of this study are available from the corresponding author upon reasonable request. Some data may not be made available because of privacy or ethical restrictions.
