# Supplementary material for: AFM-based nanoindentation indicates an impaired cortical stiffness in the AAV-PCSK9DY atherosclerosis mouse model
Source: Pflugers Arch. 2022 Jun 1;474(9):993–1002. doi: 10.1007/s00424-022-02710-x (PMC9393126; doi:10.1007/s00424-022-02710-x)
Supplement: Supplementary file 1 — Supplementary file1 (PDF 784 KB) [file 424_2022_2710_MOESM1_ESM.pdf]

AFM-based nanoindentation indicate an impaired cortical stiffness in the AAV-PCSK9DY atherosclerosis mouse model

Leonie Achner[1*], Tobias Klersy[1*], Benedikt Fels[4], Tobias Reinberger[3,7], Cosima X. Schmidt[5], Natalie Groß[8], Susanne Hille[3,6], Oliver J. Müller[3,6], Zouhair Aherrahrou[3,7], Kristina Kusche-Vihrog[3,4*], Walter Raasch[1,2,3]

[1]Institute of Experimental and Clinical Pharmacology and Toxicology, University of Lübeck, Germany, [2]CBBM (Centre for Brain, Behavior and Metabolism), University of Lübeck, Germany, [3]DZHK (German Centre for Cardiovascular Research), partner site Hamburg/Kiel/Lübeck, Germany; [4]Institute for Physiology, University Lübeck, [5]Institute of Neurobiology, University of Lübeck, Germany. [6]Department of Internal Medicine III, University Hospital Schleswig-Holstein, Campus Kiel, [7]Institute for Cardiogenetics, University Lübeck; [8]Institute for Experimental Dermatology (LIED) University of Lübeck, Germany

[*]contribute equally

Running title: Endothelial function in atherosclerosis

Corresponding author:

Walter Raasch, Ph.D, Institute of Experimental and Clinical Pharmacology and Toxicology, University of Lübeck, Ratzeburger Allee 160, 23538 Lübeck, Germany, phone: ++49-451-31017229, fax: ++49-451-31017204, e-mail walter.raasch@uni-luebeck.de

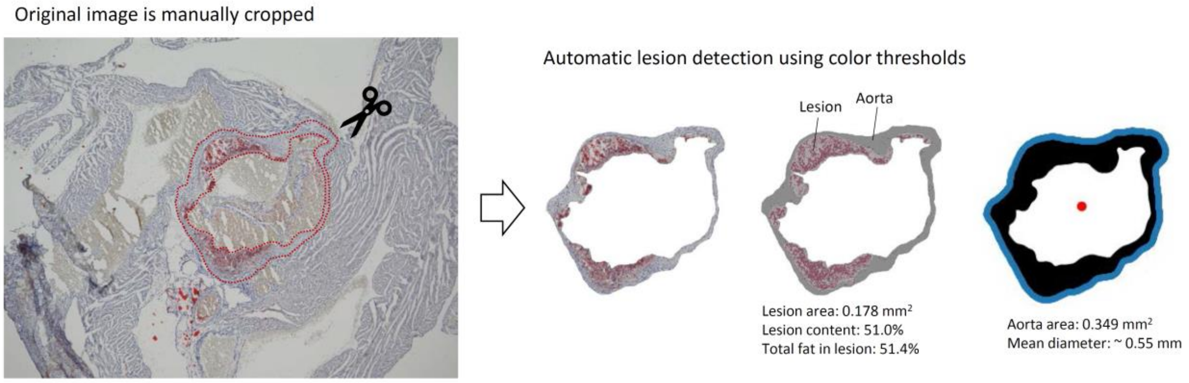

Supplementary figure S1: Workflow to quantify murine aortic lesions. Areas of lesions and OilredO-positive regions were determined using an in-house Python script (available on request). In brief, the Python package OpenCV (https://pypi.org/project/opencv-python/) was utilized to process cropped images (step 1) and to determine lesions based on color thresholds (e.g., reddish pixel for ORO).The ratio of lesions in each animal was determined as the percentage lesion area, normalized to the total area of the aorta.

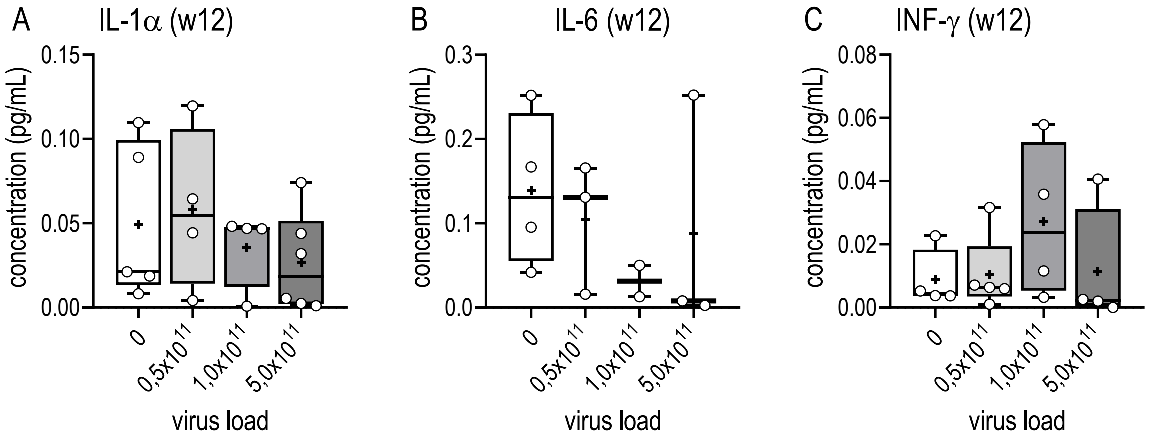

Supplementary figure S2: Fig. 2: Plasma concentrations of the cytokines IL-1α (A), IL-6 (B), and IFN-γ (C) after 12 weeks in WD-fed mice in dependency of the PCSK9 viral load. A Kruskal Wallis Test (IL-1α P=0.519; IL-6 P=0.592; INF- γ P=0396) followed by Dunn's multiple comparisons test was calculated for the various cytokines when Gaussian distribution of the values was not given. n=5-6; the median is depicted in box blots; the box extends from the 25th to 75th percentiles and the whiskers go down to the smallest value and up to the largest.
